# Evaluation of a tailored intervention to improve management of overweight and obesity in primary care: study protocol of a cluster randomised controlled trial

**DOI:** 10.1186/1745-6215-15-82

**Published:** 2014-03-19

**Authors:** Jane Krause, Shona Agarwal, Danielle H Bodicoat, Arne Ring, David Shepherd, Stephen Rogers, Michel Wensing, Richard Baker

**Affiliations:** 1 Department of Health Sciences, College of Medicine, Biological Sciences and Psychology, University of Leicester, 22-28 Princess Road West, LE1 6TP Leicester, UK; 2 Diabetes Research Centre, College of Medicine, Biological Sciences and Psychology, Leicester General Hospital, Leicester LE5 4PW, UK; 3 Leicester Clinical Trials Unit, College of Medicine, Biological Sciences and Psychology, Leicester General Hospital, Leicester LE5 4PW, UK; 4 Department of Mathematical Statistics and Actuarial Sciences,, University of the Free State, P.O. Box 339, Bloemfontein 9300, South Africa; 5 Saffron Group Practice, 509 Saffron Lane, Leicester LE2 6UL, UK; 6 Public Health Department, Guildhall Road, Northampton NN1 5DN, UK; 7 Scientific Institute for Quality of Healthcare, Radboud University Nijmegen Medical Centre, Postbus 9101, 6500 HB Nijmegen, Netherlands

**Keywords:** Obesity, Overweight, Tailored implementation of chronic diseases, TICD, Primary care teams

## Abstract

**Background:**

In the UK around 22% of men and 24% of women are obese, and there are varying but worrying levels in other European countries. Obesity is a chronic condition that carries an important health risk. National guidelines, for use in England, on the management of people who are overweight or obese have been published by the National Institute for Health and Clinical Excellence (NICE, 2006). NICE recommendations for primary care teams are: determine the degree of overweight and obesity; assess lifestyle, comorbidities and willingness to change; offer multicomponent management of overweight and obesity; referral to external services when appropriate. This study investigates a tailored intervention to improve the implementation of these recommendations by primary care teams.

**Methods/Design:**

The study is a cluster randomised controlled trial. Primary care teams will be recruited from the East Midlands of England, and randomised into two study arms: 1) the study group, in which primary care teams are offered a set of tailored interventions to help implement the NICE guidelines for overweight and obesity; or 2) the control group in which primary care teams continue to practice usual care. The primary outcome is the proportion of overweight or obese patients for whom the primary care team adheres to the NICE guidelines. Secondary outcomes include the proportion of patients with a record of lifestyle assessment, referral to external weight loss services, the proportion of obese patients who lose weight during the intervention period, and the mean weight change over the same period.

**Discussion:**

Although often recommended, the methods of tailoring implementation interventions to account for the determinants of practice are not well developed. This study is part of a programme of studies seeking to develop the methods of tailored implementation.

**Trial registration:**

Current Controlled Trials ISRCTN07457585. Registered 09/08/2013. Randomisation commenced 30/08/2013.

## Background

### Obesity

National guidelines, for use in England, on the management of people who are overweight or obese have been published by the National Institute for Health and Clinical Excellence (NICE) [1]. In the guidelines, overweight is defined as body mass index (BMI) of between 25 and 30 kg/m^2^ and obesity is defined as a BMI of 30 kg/m^2^ or over. In 2011, 65% of adult men in England were classified as overweight with 24% obese, with similar percentages among women (58% and 26% respectively) [2]. The prevalence of obesity varies by level of social deprivation, and reaches 33% in women in the lowest socio-economic group [3]. High though these levels of obesity may be, it has been predicted on the basis of current trends that they will have risen even further by 2030 [4].

Obesity is a chronic condition that confers an important health risk [5]. It has been estimated that the effects of obesity exceed those of smoking or excess alcohol [6]. It is associated with increased rates of mortality, diabetes, coronary heart disease, hypertension, osteoarthritis and some cancers, and leads to increased health care costs [7]. It also reduces health-related quality of life and increases time lost from work [4,8].

In 2011, the government in England launched a strategy intended to reduce levels of obesity by 2020 including plans to improve food labelling and encourage more healthy lifestyles [9]. Health services also have responsibilities in reducing the number of people who are overweight and obese, and excess weight in both adults and children has been included among the new outcome indicators for the English health service [10].

In England, primary care services are important in identifying people who are obese, or at risk of becoming obese, and offering advice, support, and practical interventions. Some people may be referred to receive dietary and exercise advice, or for consideration of bariatric surgery. Patients may also opt to attend weight reduction services that charge a fee, for example *Weight Watchers* or *Slimming World *[11,12]. NICE identifies a range of barriers to following the guidelines, including perceptions among patients, social pressures including marketing of foods, and poor access to weight reduction services, including exercise facilities. The most commonly reported barriers to effective management of obesity in the primary care setting include: psychological complexities of cases, high rate of relapse, perceived lack of effective interventions, lack of time, lack of resources, and lack of onward referral options [13]. In pilot work in Leicester, UK, we have identified a sense of helplessness among health professionals as a potential barrier [14]. A 2010 review of strategies to promote weight reduction in obese patients through changing the behaviour of health professionals and the organisation of care by Flodgren and colleagues [15] has identified the need for better developed and evaluated interventions to achieve effective weight reduction advice by health care professionals.

### Targets for improvement

This study is part of the Tailored Implementation for Chronic Diseases (TICD) project, which has the overall aim of developing and testing methods of tailoring implementation interventions to determinants of practice for knowledge in chronic illness care [16]. In the TICD project, we developed a programme for implementing the NICE obesity guidelines in a stepwise approach. The first step involved identifying from the NICE guidelines those recommendations applicable to primary care, and which would be selected for implementation (see Table 1) [1].

**Table 1:** The NICE recommendations for the treatment of overweight and obesity

	**Recommendation**	
1	Determining degree of overweight and overweight	• Use clinical judgement to decide when to measure weight and height
		• Use body mass index to classify the degree of overweight or obesity
		• Use waist circumference in people with a body mass index less than 35 kg/m^2^ to assess health risks
		• To tell the patient their classification, and how this affects their risk of long-term health problems
2	Assessment of lifestyle and willingness to change	• Presenting symptoms and underlying causes of overweight or obesity
		• Risk factors and comorbidities
		• Eating behaviour, diet and physical activity
		• Willingness and motivation to change
3	Management of overweight and obesity	• Increased physical activity
		• Improved eating behaviour
		• Healthy eating
		• If appropriate, drug treatment
4	Referral	• For assessment of the underlying cause of overweight or obesity
		• If conventional treatment has failed
		• If specialist interventions may be needed

### Health system context

England has a national health service that provides free access to care, and is funded from taxation. However, there are charges for a limited range of services, including dental care, and patients aged under 60 are required to a pay fixed charge for prescription medicines. Following organisational reforms in 2013, public health services are managed through local authorities or councils. Hospitals provide inpatient and outpatient care, and primary health care is delivered through general practices. Around 98% of the population is registered with a general practice, practices having registered lists of patients, electronic record systems, and multidisciplinary primary care teams that include doctors, nurses, and health care assistants [17]. Practices are paid through a combination of capitation and pay for performance schemes, notably the quality and outcomes framework (QOF) and payments for enhanced services. Practices vary widely in size, although the mean size is approximately 6,800 patients [17]. In 2012, there were 8,088 general practices in England, 921 of which were classified as single-handed. There were 35,871 full time equivalent general practitioners (GPs), 14,695 full time equivalent practice nurses, and 70,851 full time equivalent practice staff in other categories. The total number of patients registered with GPs in England was 55.7 million (patients are required to register with a single practice in order to receive primary care).

### Usual care

There is an indicator for obesity in the QOF as follows: the contractor establishes and maintains a register of patients aged 16 or over with a BMI ≥30 in the preceding 12 months, and therefore practices have some financial incentive for the detection of obesity [18]. Yet despite this, the proportion of the population recorded on general practice obesity registers falls well short of the proportion identified as obese in systematic population surveys, nationally being only 10.7% in 2012 [19] compared with approximately 20% in population surveys.

Failure to record obesity is not the only performance deficit. An implementation uptake report on the surgical and pharmacological interventions for obesity found that the NICE clinical guidelines had mixed impact in practice [1]. Prescription patterns were in line with the NICE guidance but the low proportion of patients receiving advice prior to the start of their drug treatment indicated deviance from the guidance. Lack of adherence to the NICE clinical guidelines for obesity was also reported by the Office of Health Economics [20]. The Office of Health Economics, which administered questionnaires to the 151 Primary Care Trusts that managed primary care teams in England at the time of the survey to assess their views on the degree of similarity between their local referral process/guidelines for obesity and the NICE guidance. People with obesity, therefore, may well not be identified and recorded in general practice, and, even if they are, support and management may not be in accordance with the guidelines. Yet, with around a quarter of the UK population being obese [17], the effective implementation of the NICE clinical guidance on obesity has potential to benefit individual and population health and reduce the economic burden of the health consequences of obesity [1].

### The Tailored Implementation for Chronic Diseases project

The underlying conceptual framework to TICD is that implementation is more likely to be effective when the determinants of practice have been identified and a strategy (that may include several discrete interventions) is designed and delivered to address them. This process is referred to as tailored implementation.

Our Cochrane review [21] has shown that this approach can be effective, but as yet the most appropriate methods used to identify determinants and tailor a strategy to account for them have not been determined. In the review, twelve studies provided sufficient information to be included in the quantitative analysis, and a pooled odds ratio of the effectiveness of tailored interventions was 1.54 (95% confidence interval 1.16 to 2.01). TICD sets out to advance the methodology of tailored implementation.

The first component study of TICD, a review of determinants and development of a checklist that can assist in identifying them in implementation initiatives, has been completed and published [22]. A second study has compared various methods for investigating determinants; for example, interviews of professionals or patients, or brainstorming (manuscript in preparation). A third study has investigated the methods that can be used to plan the implementation strategy taking account of the determinants of practice (manuscript in preparation). Both the second and third studies have been completed, and have laid the foundation for the trial described in this protocol.

### Objectives

The primary aim of the study is to examine the effectiveness of a tailored implementation strategy in comparison with usual care for improving adherence to the NICE guidelines for the management of overweight and obesity in primary care teams.

The secondary aim is to examine the extent to which the most important determinants were identified and appropriate interventions used to address them, which we refer to as the validity of methods. A process evaluation will be undertaken to investigate how successful the tailoring process was in identifying and addressing determinants, the fidelity of intervention delivery, and how the study participants responded to the intervention.

### Research questions

1. What is the effectiveness of a tailored implementation programme compared to no intervention (usual care) in improving adherence of primary care teams to the NICE guidelines on overweight and obesity?

2. What is the validity of the methods used to tailor the implementation programme to determinants of practice?

## Methods/Design

### Trial design

This study is a cluster randomised trial in primary care [23,24] that will randomise general practices into two study arms: 1) the study group in which primary care teams are offered a set of tailored interventions; or 2) the control group in which primary care teams administer usual care.

### Participants and setting

All general practices in the East Midlands of England will be invited to participate in the study. The area includes the following Clinical Commissioning Groups (CCGs): Leicester City CCG, East Leicester & Rutland CCG, West Leicestershire CCG, NHS Southern Derbyshire CCG, North Derbyshire CCG, NHS Lincolnshire East CCG, Lincolnshire West CCG, North East Lincolnshire CCG, North Lincolnshire CCG, Mansfield and Ashfield CCG, Nottingham North & East CCG, Nottingham West CCG, Rushcliffe CCG, NHS Nottingham City CCG, Newark & Sherwood CCG, NHS Corby CCG, Nene CCG. This is an area of approximately 3.5 million inhabitants, served by 630 general practices. Further information on the demographic and disease prevalence within the CCGs is shown in Table 2. The population is typical of England for social status and age distribution, but with a slightly higher proportion of non-white ethnicity. There are three to four large urban centres, but also small towns and more isolated rural communities. Levels of obesity are around the national average (25%), and obesity is a key regional health priority [25]. In recruiting primary care teams, we will work with the East Midlands Primary Care Research Network.

**Table 2:** **Demographic and disease prevalence within Clinical Commissioning Groups **[26,27]

**Clinical Commissioning Group (CCG)**	**Average index of multiple deprivation IMD 2010 score**	**Population density (persons per hectare)**	**Population from black ethnic groups**	**Population from Asian ethnic groups**	**Disease prevalence (number and percentages); that is, patients on general practice quality and outcomes framework registers with these conditions**		
					**Obesity**	**Coronary heart disease**	**Diabetes mellitus**
Corby CCG	26.49	7.6	1.6%	1.3%	4,869 (11.9%)	1,732 (3.4%)	2,119 (5.2%)
East Leicester and Rutland CCG	9.96	2.0	0.7%	7.2%	24,258 (9.4%)	10,786 (3.4%)	13,555 (5.3%)
Leicester City CCG	33.68	45.0	6.2%	37.1%	28,405 (9.8%)	10,018 (2.7%)	19,959 (7.0%)
Lincolnshire West CCG	19.29	1.9	0.5%	1.3%	22,375 (12.2%)	9,163 (4.1%)	10,219 (5.7%)
Lincolnshire East CCG	24.14	0.9	0.3%	0.8%	12,675 (5.3%)	24,711 (12.2%)	14,784 (7.4%)
Mansfield and Ashfield CCG	29.32	11.3	0.4%	1.1%	21,473 (14.3%)	7,887 (4.3%)	8,714 (5.9%)
Nene CCG	16.92	3.1	2.6%	4.0%	50,416 (10.1%)	18,977 (3.1%)	26,742 (5.4%)
Newark & Sherwood CCG	19.05	1.8	0.3%	0.9%	13,354 (12.7%)	5,168 (4.1%)	5,563 (5.4%)
North Derbyshire CCG	17.84	2.4	0.4%	1.0%	26,401 (11.0%)	12,345 (4.3%)	14,307 (6.0%)
North East Lincolnshire CCG	29.77	8.3	0.3%	1.3%	17,943 (13.2%)	7,101 (4.3%)	8,227 (6.2%)
North Lincolnshire CCG	22.21	2.0	0.3%	2.7%	18,424 (13.4%)	7,681 (4.6%)	8,582 (6.3%)
Nottingham City CCG	35.48	41.0	7.3%	13.1%	27,070 (9.7%)	9,675 (2.8%)	13,328 (4.9%)
Nottingham North & East CCG	17.46	9.3	1.3%	2.5%	6,741 (5.8%)	5,635 (3.9%)	13,039 (11.0%)
Nottingham West CCG	14.41	13.7	0.9%	4.1%	8,271 (10.7%)	3,826 (4.1%)	4,178 (5.5%)
Rushcliffe CCG	7.62	2.7	0.6%	4.2%	8,489 (8.5%)	4,375 (3.6%)	4,373 (4.4%)
Southern Derbyshire CCG	19.96	4.7	1.6%	6.8%	47,205 (11.1%)	18,745 (3.6%)	26,086 (6.2%)
West Leicestershire	13.09	4.2	0.5%	4.9%	29,878 (9.9%)	11,790 (3.2%)	16,329 (5.5%)

#### *Patients*

Eligible patients are adults aged 16 years and over in the participating practices who are either overweight or obese, whether or not they are recorded in the practice obesity register. Patients will not be randomised or directly involved in the study, although anonymised data will be extracted from their electronic medical records. Overweight is defined as a BMI of between 25 and 30 kg/m^2^ and obesity is defined as a BMI of over 30 kg/m^2 ^[1].

### Implementation programme

#### *Development of the implementation programme*

In previous phases of the TICD project (reported in detail elsewhere [16]), determinants for the implementation of the targeted recommendations have been identified as well as strategies for identifying those determinants. Samples of implementation experts, health professionals and patients were involved in a variety of methods to collect information on determinants (brainstorming, structured group discussion, interviews and questionnaires). The identified determinants were assessed for their importance in determining practice (important determinants were those that had a large impact on practice), and were capable of being addressed (it was plausible that the determinant could be addressed to improve clinical practice). Those determinants judged plausibly important were then subjected to a systematic process for selecting interventions. New samples of implementation experts, professionals and patients were involved in group meetings, and asked to propose interventions that would be likely to address the determinants (see Table 2). On the basis of this prior work a tailored implementation programme has been developed, in which each intervention addresses one or more specific determinants. These are outlined in Table 3.

**Table 3:** Interventions that address determinants

	**Recommendation**	**Determinants**	**Interventions**
1	Determining degree of overweight and overweight	Acceptable ways to raise and discuss the issue with patients	Training and model scripts on discussing weight with patients
		How to effectively measure waist circumference	Training in waist measurement
2	Assessment of lifestyle and willingness to change	Ways to assess willingness to change	Training
		Resources to motivate and inform	Booklet for patients
3	Management of overweight and obesity	Lack of prescriptive information	Training and booklet
		Lack of knowledge	Discussion with practices on delegation and the role for practice nurses
4	Referral	Lack of information on referral pathways	Provision of information on local referral pathways

**Figure 1:**
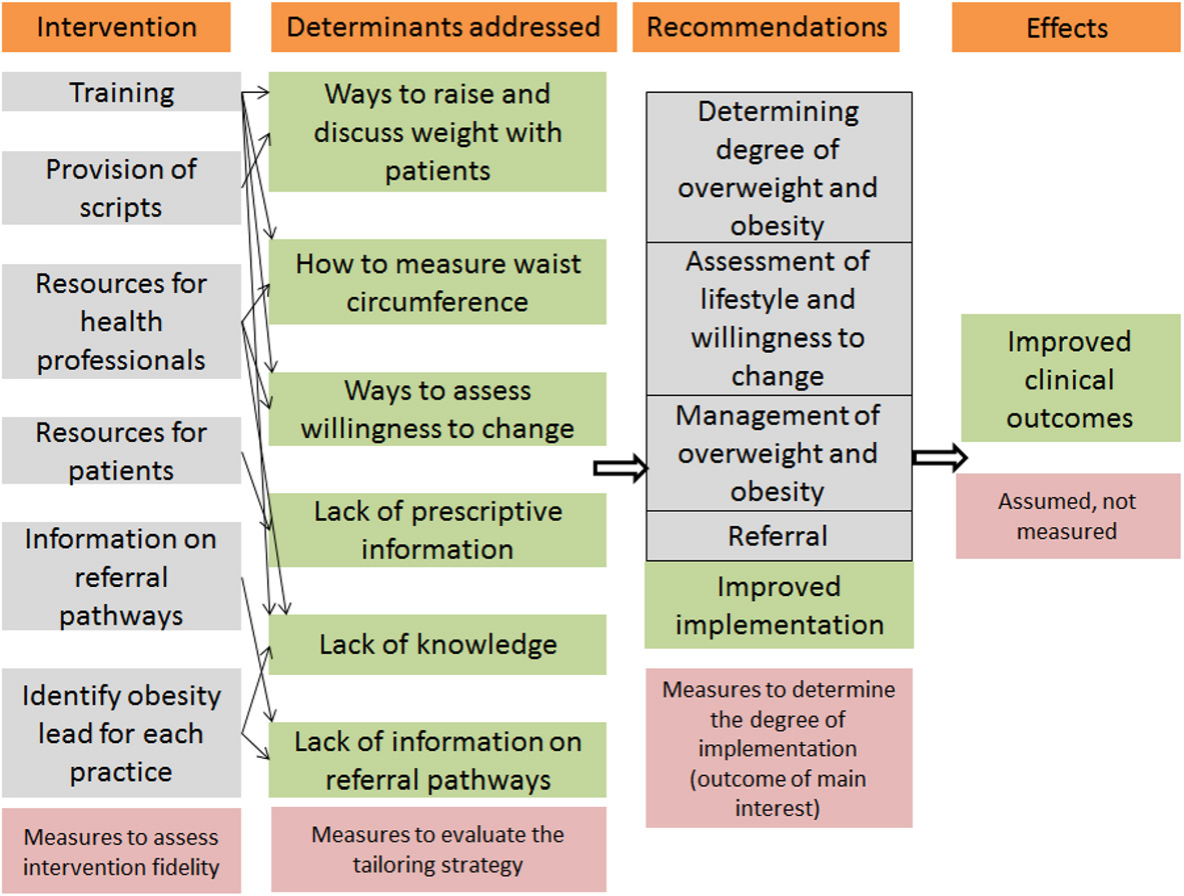
**An outline of the logic model of the intervention.** We aim to measure the following determinants of change: a) the capacity of each practice – and to classify practices according to their resources and ability to implement an intervention, the average number of patients per general practitioner; b) engagement with the intervention – scaling and recording (measured by observations and example patients); and c) identify what practices did as a result of the intervention and why – measured by interviewing one or two members of the practice.

Delivering the interventions will involve visits to practices and interaction with the primary care teams (GPs and practice nurses). The interventions will be delivered to as many members of the primary care team as possible. We will also ask for one member of the team (usually a practice nurse) to take the lead on the improvement of overweight and obesity care within their team. We will work with the identified person to improve their knowledge and resources to effectively manage the care of their overweight and obese patients (Figure 1).

#### *Logic model*

**Figure 2:**
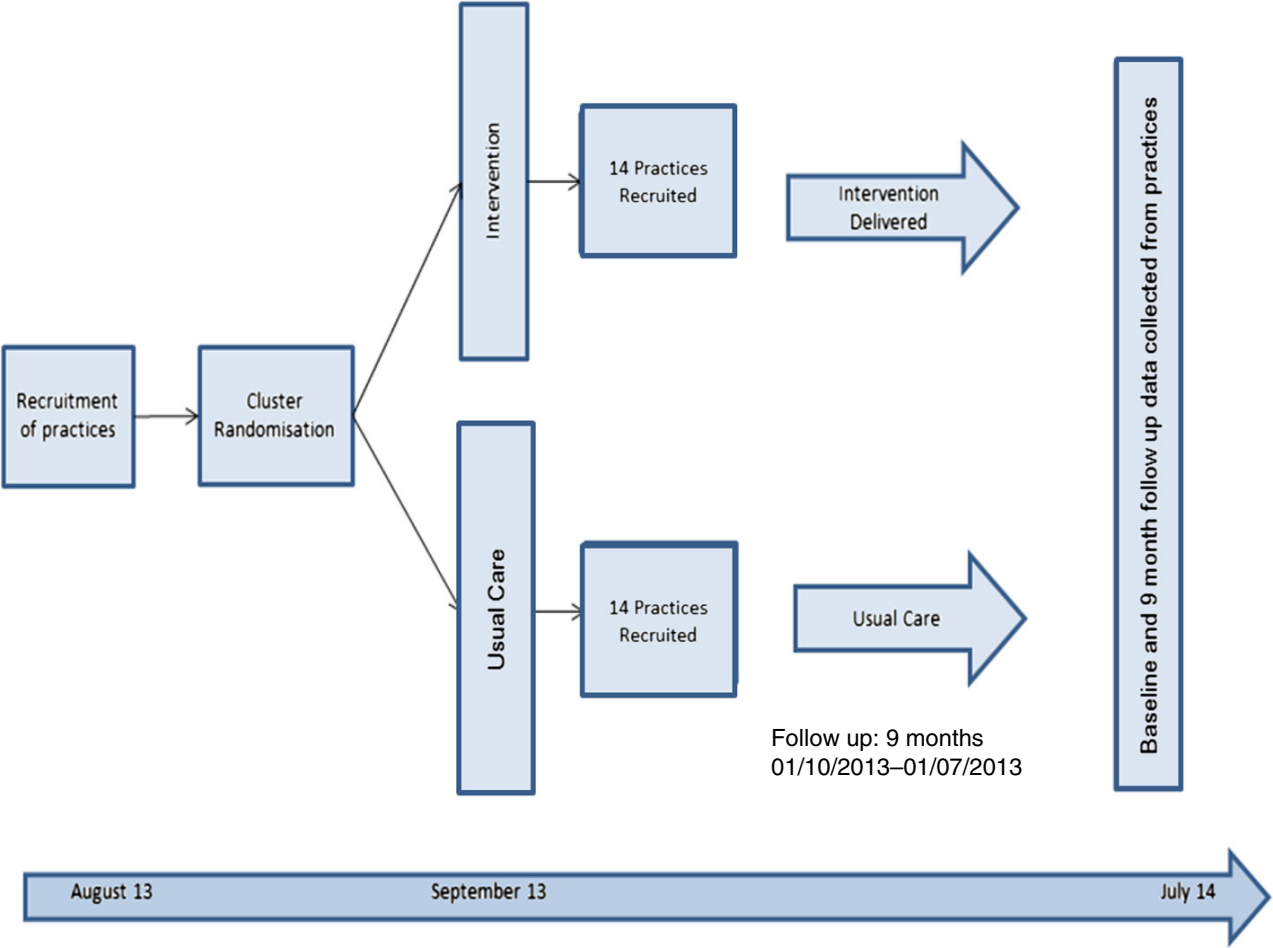
A flow diagram and timescale of the study.

The interventions (which aim to address the determinants listed in Table 3) will include a practice-based, interactive session which will be delivered to as many members of the recruited primary care teams as possible (Figure 2). The session will provide teams with a reminder of the NICE guidelines for overweight and obesity, and with practical tips and suggestions for their implementation within their clinical practice. The session will be delivered primarily as a presentation plus interactive discussion focused around each of the four NICE recommendations and tailored to address the determinants identified in study 2.

We will also offer the study teams repeat visits, monthly telephone calls with the obesity lead, and a closed list of contacts to enable them to establish a support network with other study group teams.

#### *The recommendations*

The implementation programme will address the determinants relating to each of the four targeted guideline recommendations.

##### 

**Recommendation 1: Determining the degree of overweight and obesity** Determinants addressed:

• Acceptable ways to raise and discuss the issue of weight with patients

• How to effectively measure waist circumference

The issue of raising weight with patients is complex and, if not raised sensitively, patients can be resistant to discuss their weight or follow a proposed weight loss intervention. Study 2 confirmed that health care professionals found it difficult to find acceptable ways to raise the issue of weight with patients. The intervention aims to address this by providing scripts for health care professionals to use in raising and discussing weight with patients that are likely to be acceptable to patients. We also aim to show video clips of good and bad ways to raise weight with patients.

To classify the degree of overweight and obesity, the NICE guidelines recommend that waist circumference is measured in patients with a BMI less than 35 kg/m^2^ to assess health risks. Study 2 identified that health care professionals were unsure how to reliably measure waist circumference and how waist circumference relates to health risks. The intervention aims to address this by providing information on how to effectively measure waist circumference. A live demonstration will be provided in the practices and a thorough explanation relating waist circumference and increased health risks will be provided. In addition health care professionals will also be provided with posters (containing information on how to measure waist circumference) which can be placed in clinic rooms to provide a visual guide for them to follow while measuring waist circumference.

##### 

**Recommendation 2: Assessment of lifestyle and willingness to change** Determinants addressed:

• Ways to assess willingness to change

• Resources to motivate and inform patients

In order for a weight loss intervention to be successful the patient needs to be ready and willing to make changes to their lifestyle. If the patient is not ready and willing to do so, it is likely the intervention will fail. The NICE guidelines recommend that health care professionals assess a patient’s lifestyle, co-morbidities and willingness to change. This can be difficult to assess in a short consultation. The intervention will explain how to assess whether a patient is ready to make changes to their lifestyle. Health care professionals will also be provided with questionnaires to assess motivation and that can be used as a discussion point with patients.

The intervention will also provide health care professionals with resources that can be used to increase a patient’s motivation, and suggest appropriate times to use them within the consultation. The resources will demonstrate the benefit of a modest weight loss (5 or 10% of body weight) for the patient.

##### 

**Recommendation 3: Management of overweight and obesity** Determinants addressed:

• Lack of prescriptive information

• Lack of knowledge

Many patients interviewed during our investigation of determinants felt that they were aware of the concept of healthy eating, and the need to reduce their calorie intake to lose weight, but they felt more prescriptive advice would be of benefit. They felt that the advice provided by commercial slimming clubs on portion sizes and guidance on a more prescriptive diet would be beneficial. The intervention will provide health care professionals with a prescriptive weight loss plan that can be delivered to patients within primary care. The ‘Weight loss you can see’ information booklet provides patients with visible pictures of portion sizes for every day foods. The booklet provides patients with a prescriptive energy deficit diet. The energy level prescribed for a patient will be based on an estimate of their initial maintenance energy needs minus 600 kcal/day [1]. Health care professionals will be provided with training to calculate energy requirements, and the required portions of carbohydrates, vegetable, protein and dairy foods [28].

Health care professionals felt that they did not always have sufficient knowledge or skill to advise patients on changes to their diet. The intervention will also provide health care professionals with a summary of the NICE guidelines for diet and exercise.

##### 

Recommendation 4: Referral

Determinants addressed:

• Lack of information on referral pathways

There are many community run programmes and initiatives in primary care to improve health and assist weight loss. Some of these programmes are available for patients to self-refer into, while others require a referral from a health care professional. Our investigation of determinants found that many health care professionals were not aware of the services on offer and how to refer patients to them. The intervention aims to provide health care professionals with an up to date list of the current services offered locally. In addition clear guidance on the referral pathway to local secondary services will also be given.

We will also ask each primary care team to designate a health care professional for their team to lead their management of overweight and obesity. We will speak with the local lead prior to delivering the intervention to identify their current treatment plan for overweight and obesity to identify any areas where the intervention may need to be further tailored to meet the needs of the individual practice. We will also encourage the local lead to take charge in implementing the intervention with their team.

The intervention given to health care professionals will be based on the NICE guidelines for obesity, and when appropriate will draw on other resources such as the NICE obesity presenter slides, the NICE obesity baseline assessment tool, *Lightening the load: tackling overweight and obesity *[29] and the obesity care pathway and *Your weight, your health *[30].

#### *Determinants not addressed by the implementation intervention*

The implementation intervention aims to address a variety of determinants identified in previous work. There were some determinants which could not be addressed within this intervention, generally because additional resources would be required. These include:

• Use of health care assistants/receptionists to weigh patients

• Patients are only weighed as part of their routine care. Therefore, some patients are not weighed on a regular basis.

• A trained specialist individual within primary care who can conduct everything (weighing, advice, assess motivation, offer referral).

• Local referral policies may restrict the range of patients who can be referred to specialist care.

• Dietitian run clinic in GP surgery.

• Running a group service at the GP surgery which provides weekly advice/support.

The teams in the control group will receive no interventions. After the trial has ended, they will receive feedback about their own performance in comparison with the performance of others in the study, in relation to the guideline recommendations. They will receive no other interventions.

If the intervention is successful, teams in the control group will be offered a compact disc containing all the materials and resources used within the intervention teams.

### Outcomes

#### *Primary outcome*

The primary outcome measure relates to recommendation 3 (Table 1), the proportion of overweight or obese patients to whom the health professional has offered a weight loss intervention within the study period. The patient population is defined as all patients with a BMI measurement of 25 kg/m^2^ or higher recorded in their medical notes at the time of the centre randomisation.

#### *Secondary outcomes*

We will collect data from each practice on:

• The proportion of patients with a BMI or waist circumference measurement recorded within the study period (recommendation 1);

• the proportion of patients with a record of lifestyle assessment (recommendation 2);

• referral to external weight loss services (recommendation 4);

• the proportion of overweight/obese patients who changed weight (lost or gained 1 kg) during the study period;

• the mean weight change over the same period.

### Measurement procedures

In each general practice, measurements of performance by participating primary care teams (GPs and practice nurses) will be performed at baseline and at follow-up after up to 9 months (the study period).

The following measurement methods will be used: record audit for primary and secondary outcome measures, and interviews and questionnaires for primary care teams to collect information on the process of the study.

Specific measures will include:

1. Data relating to the primary and secondary outcome measures will be obtained from the electronic medical records that are routinely collected in each general practice. We have developed bespoke data extraction software for use in a pilot study. This software will be adapted to ensure capture of all the relevant data from records. All adults in each practice are included, and full data protection procedures will be followed. The software will identify those patients with a recorded BMI of 25 kg/m^2^ or over, and the recorded management of those patients. Baseline and follow-up data will be collected in one data extraction at the end of the intervention.

2. An interview will be undertaken with a primary care team member to identify the numbers of registered patients, patient demography, performance in the QOF and procedures for managing obesity.

3. To assess the tailoring of the intervention, we will record notes on the delivery of the implementation interventions to each team, how it is perceived and if and how it is adapted. We will also interview a member of the primary care team to explore their experiences of the interventions.

4. The practice obesity lead will be asked to complete a self-report questionnaire to the obesity lead of the participating primary care team to identify any deviations from the interventions and any adaptations that were necessary, and why.

### Process evaluation

A detailed protocol for a process evaluation has been developed as is being reported separately [31]. In accordance with this protocol, we aim to examine the fidelity of the planned intervention strategy and how this relates to the effectiveness of the implementation programme, and to identify possible mechanisms underlying effectiveness (or lack of it) on primary and secondary outcomes.

### Recruitment

All primary care teams in the East Midlands of England will be invited to participate in the study. The area includes the following CCGs: Leicester City CCG, East Leicester & Rutland CCG, West Leicestershire CCG, NHS Southern Derbyshire CCG, North Derbyshire CCG, NHS Lincolnshire East CCG, Lincolnshire West CCG, North East Lincolnshire CCG, North Lincolnshire CCG, Mansfield and Ashfield CCG, Nottingham North & East CCG, Nottingham West CCG, Rushcliffe CCG, NHS Nottingham City CCG, Newark & Sherwood CCG, NHS Corby CCG and Nene CCG. Letters of invitation, with information about the study, will be sent to all the primary care teams in these CCGs.

### Randomisation

Randomisation to the two treatment groups will take place at the level of the practice team; however, we are undertaking the primary analysis at the patient level, and therefore this is a cluster randomised trial.

The randomisation will be stratified by list size (<6000, ≥6000) and deprivation (<20,≥20 practice Index of Multiple Deprivation scores 2010), where the cut-off points are related to the median of each of variable of all regions in England [27].

Centres will apply for randomisation sequentially. To avoid larger imbalances between the four strata (to mimic the underlying distribution of GP practices in England), the following restriction will be implemented: none of the 4 strata shall contain more than 10 practices, and the maximum of 10 practices is only allowed if all other strata contain at least 4 practices, otherwise the maximum should be 8. The randomisation will be performed independently from the trial office by the Leicester Clinical Trials Unit.

### Blinding

Participant teams cannot be blinded to whether or not they are receiving an intervention. Data collection will use a standard electronic system and, to minimise bias, all data will be collected using full anonymisation using a standardized data query.

### Statistical methods

Using an intention-to-treat approach, the primary analyses will use generalised estimating equations that assume the outcome has a binomial distribution, include a term to account for the practice-level clustering, and use a logit link function to compare the proportion of overweight and obese patients that have been offered a weight loss intervention between the two arms. The secondary outcomes will be analysed using similar methods, with appropriate distributions and link functions selected. Baseline is defined as the last value obtained within 1 year prior to randomisation. Missing values of quantitative endpoints will not be imputed.

A detailed Statistical Analysis Plan will be produced prior to collection of the data and the database lock. Data collected from electronic records will be transferred to a statistical package for analysis. A Consolidated Standards of Reporting Trials (CONSORT) diagram showing the progress of clusters and patients through phases of the trial will be produced [32]. Descriptive characteristics of the centres and their patients at baseline and follow-up will be summarized by treatment arm, using mean (standard deviation) or median (interquartile range) for continuous variables as appropriate, and count (percentage) for categorical variables.

### Sample size

It is assumed that, in the control arm, the adherence will be 46%. These estimates are based on a local pilot study of management of obesity in primary care completed in 2010 to 2011, and were measured at the team level and so are an approximation [14]. The aim of the study is to detect an increase to 60% adherence in the intervention arm with 80% power, using a two-sided test with alpha of 0.05. The intraclass correlation coefficient is assumed to be 0.05.

We determined the number of clusters per treatment using these values and with various numbers of clusters and cluster sizes (Table 4) (R-package CRTsize) [33].

**Table 4:** Study power depending on number of clusters and average cluster size

Average cluster size (M)	> 1,130	163-212	213-309	310-568	569-3,464	> 3,464	> 287
Power (1-β)	0.80	0.80	0.81	0.82	0.83	0.84	0.85
Number of clusters (N)	12	13					14

As the average number of overweight patients per centre with baseline data is likely to be larger than about 500, it was deemed adequate to choose a total sample size of 28 primary care teams, which would allow adequate power even in the case of a drop out of up to four teams.

### Ethics

Research ethical approval was granted from the NRES Committee London - Camden & Islington (13/LO/1157) on 18 July 2013.

## Discussion

This study is part of a programme of research being undertaken in five European countries to develop the methods of tailored implementation [16]. A rigorous process has been followed to select approaches for identifying the determinants of practice [22], and a systematic procedure was followed to apply methods of selecting interventions to account for the determinants. Obesity is a serious problem for health services, and it is not always identified and managed effectively by primary care services in England and other countries. Therefore, in addition to providing evidence about the methods of tailoring, the study should improve evidence on approaches to improving the care of obese patients in primary care.

## Trial status

Invitations to participate in the study have been sent to practices. Randomisation of recruited practices has commenced.

## Abbreviations

BMI: Body mass index; CCG: Clinical Commissioning Group; CONSORT: Consolidated Standards of Reporting Trials; GP: general practitioner; NICE: National Institute for Health and Clinical Excellence; QOF: quality and outcomes framework; TICD: Tailored Implementation for Chronic Diseases.

## Competing interests

The authors declare that they have no competing interests.

## Authors’ contribution

JK, RB and SA conceived the trial and have been involved in all stages of the study design, and together with DHB, AR, DS, SR and MW participated in writing the protocol and submission to the ethics committee. DB and AR are the trial statisticians. DS created the search query for the data extraction. SR and MW critically assessed the study protocol. All authors approved the final manuscript. This study protocol has been discussed with and reviewed by the project partners.

## References

[B1] NICE Obesity: Guidance on the Prevention, Identification, Assessment and Management of Overweight and Obesity in Adults and Children 2006 London: National Institute for Health and Clinical Excellence 22497033

[B2] Health Survey for England Health, Social Care and Lifestyles: 2011 http://www.hscic.gov.uk/catalogue/PUB09300 (accessed 15 July 2013)

[B3] Review M Fair Society, Healthy Lives 2010 London: The Marmot Review

[B4] Wang YC McPherson K Marsh T Gortmaker SL Brown M Health and economic burden of the projected obesity trends in the USA and the UK Lancet 2011 378 815 825 10.1016/S0140-6736(11)60814-3 21872750

[B5] Gaziano JM Fifth phase of the epidemiologic transition: the age of obesity and inactivity JAMA 2010 303 275 276 10.1001/jama.2009.2025 20071469

[B6] Sturm R The effects of obesity, smoking, and drinking on medical problems and cost Health Aff 2002 21 Suppl 2 245 253 10.1377/hlthaff.21.2.245 11900166

[B7] Finkelstein EA Trogdon JG Cohen JW Dietz W Annual medical spending attributable to obesity: payer-and service-specific estimates Health Aff 2009 28 Suppl 5 822 831 10.1377/hlthaff.28.5.w822 19635784

[B8] Larsson U Karlsson J Sullivan M Impact of overweight and obesity on health-related quality of life - a Swedish population study Int J Obes 2002 26 417 424 10.1038/sj.ijo.0801919 11896499

[B9] Department of Health Healthy Lives, Health People. A Call to Action on Obesity in England 2011 London: Department of Health

[B10] Department of Health Improving Outcomes and Supporting Transparency. Part 1A: A Public Health Outcomes Framework for England 2012–2016 2012 London: Department of Health

[B11] Weight Watchers http://www.weightwatchers.com (accessed 22 July 2013)

[B12] Slimming world http://www.slimmingworld.com (accessed 22 July 2013)

[B13] Maryon-Davis A Weight management in primary care: how can it be made more effective? Proceed Nutr Soc 2005 64 97 103 10.1079/PNS2004414 15877928

[B14] Gunther S Guo F Sinfield P Rogers S Baker R Barriers and enablers to managing obesity in general practice: a practical approach for use in implementation activities Qual Primary Care 2012 20 93 103 22824562

[B15] Flodgren G Deane K Dickinson HO Kirk S Alberti H Beyer FR Brown JG Penney TL Summerbell CD Eccles MP Interventions to change the behaviour of health professionals and the organisation of care to promote weight reduction in overweight and obese adults Cochrane Database Syst Rev 2010 3 CD000984 10.1002/14651858.CD000984.pub2 PMC4235843 20238311

[B16] Wensing M Oxman A Baker R Godycki-Cwirko M Flottorp S Szecsenyi J Grimshaw J Eccles M Tailored Implementation for Chronic Diseases (TICD): a project protocol Implement Sci 2011 6 103 10.1186/1748-5908-6-103 21899753 PMC3179734

[B17] Statistics on Obesity, Physical Activity and Diet: England 2010 http://www.hscic.gov.uk/pubs/opad10 (accessed 6 February 2011)

[B18] Employers NHS 2013/14 General Medical Services (GMS) Contract and Quality and Outcomes Framework (QOF). Guidance for GMS Contract 2013 London: NHS Employers, General Practitioners Committee, NHS England

[B19] Health & Social Care Information Centre Quality and Outcomes Framework - 2011–12, England level 2012 http://www.hscic.gov.uk/catalogue/PUB08661 (accessed 23 July 2013)

[B20] Office of Health Economics (OHE) Shedding the Pounds: Obesity management, NICE guidance and bariatric surgery in England 2010 London: Office of Health Economics (OHE) http://www.ohe.org/publications/article/shedding-the-pounds-obesity-management-in-england-16.cfm (accessed 6 February 2011)

[B21] Baker R Camosso-Stefinovic J Gillies C Shaw EJ Cheater F Flottorp S Robertson N Tailored interventions to overcome identified barriers to change: effects on professional practice and health care outcomes Cochrane Database Syst Rev 2010 3 CD005470 10.1002/14651858.CD005470.pub2 PMC4164371 20238340

[B22] Flottorp SA Oxman AD Krause J Musila NR Wensing M Godycki-Cwirko M Baker R Eccles MP A checklist for identifying determinants of practice: a systematic review and synthesis of frameworks and taxonomies of factors that prevent or enable improvements in health care professional practice Implement Sci 2013 8 1 11 10.1186/1748-5908-8-1 23522377 PMC3617095

[B23] Eccles M Grimshaw J Campbell M Ramsay C Grol R, Baker R, Moss F Research designs for studies evaluating the effectiveness of change and improvement strategies. Chapter 7 Quality Improvement Research 2004 London: BMJ Books 97 114 10.1136/qhc.12.1.47 PMC1743658 12571345

[B24] Donner A Klar N Design and Analysis of Cluster Randomisation Trials in Health Research 2000 London: Arnold

[B25] East Midlands Academic Health Science Network Prospectus 2013 http://www.clahrc-ndl.nihr.ac.uk/clahrc-ndl-nihr/publications/emahsn-prospectus.aspx (accessed 15 August 2013)

[B26] NHS England CCG and Local Authority Information Packs 2013 Available at: http://www.england.nhs.uk/la-ccg-data/ (Accessed 19 August 2013)

[B27] Department for Communities and Local Government Indices of Deprivation 2010 https://www.gov.uk/government/uploads/system/uploads/attachment_data/file/6875/1871537.xls (accessed 18 August 2013)

[B28] Nutrition and Diet Resources UK’Weight loss you can see’ 2011 Glasgow, UK: Nutrition and Diet Resources UK

[B29] National Heart Forum Lightening the Load, Tackling Overweight and Obesity. A Toolkit for Developing Local Strategies to Tackle Overweight and Obesity in Children and Adults 2007 London: National Heart Forum in association with Faculty of Public Health and Department of Health http://www.nwhpaf.org.uk/downloads/7768317074796826017122007115837.pdf (accessed 15 August 2013)

[B30] Department of Health and Central Office of Information Your Weight, Your Health 2006 http://webarchive.nationalarchives.gov.uk/+/dh.gov.uk/en/publicationsandstatistics/publications/publicationspolicyandguidance/dh_4134408 (accessed 17 August 2013)

[B31] Jager C Freund T Steinhauser J Aakhaus E Flottorp S Godycki-Cwirko M Lieshout J Krause J Szecsenyi J Wensing M Tailored implementation for chronic diseases (TICD): a protocol for process evaluation in five cluster randomized controlled trials in five European countries Trials 2014 15 87 10.1186/1745-6215-15-87 24655439 PMC3994491

[B32] Schulz KF Altman DG Moher D for the CONSORT Group CONSORT Statement: updated guidelines for reporting parallel group randomised trials BMJ 2010 340 332 10.1136/bmj.c332 20619135

[B33] Package ‘CRTSize’ 2013 http://cran.r-project.org/web/packages/CRTSize/index.html (accessed 19 August 2013)

